# Implications of Standardized Uptake Values of Oral Squamous Cell Carcinoma in PET-CT on Prognosis, Tumor Characteristics and Mitochondrial DNA Heteroplasmy

**DOI:** 10.3390/cancers13092273

**Published:** 2021-05-10

**Authors:** Lukas Latzko, Bernd Schöpf, Hansi Weissensteiner, Federica Fazzini, Liane Fendt, Eberhard Steiner, Emanuel Bruckmoser, Georg Schäfer, Roy-Cesar Moncayo, Helmut Klocker, Johannes Laimer

**Affiliations:** 1 University Hospital for Craniomaxillofacial and Oral Surgery, Medical University of Innsbruck, A-6020 Innsbruck, Austria; lukas.latzko@tirol-kliniken.at; 2 Institute of Genetic Epidemiology, Department of Genetics and Pharmacology, Medical University of Innsbruck, A-6020 Innsbruck, Austria; bernd.schoepf@gmail.com (B.S.); hansi.weissensteiner@i-med.ac.at (H.W.); federica.fazzini@gmail.com (F.F.); liane.fendt@i-med.ac.at (L.F.); 3 Division of Experimental Urology, Department of Urology, Medical University of Innsbruck, A-6020 Innsbruck, Austria; eberhard.steiner@tirol-kliniken.at (E.S.); helmut.klocker@i-med.ac.at (H.K.); 4 Oral and Maxillofacial Surgeon, Private Practice, A-5020 Salzburg, Austria; research@bruckmoser.info; 5 Institute for Pathology, Neuropathology and Molecular Pathology, Medical University of Innsbruck, A-6020 Innsbruck, Austria; georg.schaefer@i-med.ac.at; 6 University Hospital of Nuclear Medicine, A-6020 Innsbruck, Austria; anmeldung@womed.at

**Keywords:** oral squamous cell carcinoma, ^18^[F]FDG-PET-CT, standardized uptake values, mitochondrial DNA heteroplasmy

## Abstract

**Simple Summary:**

In this study, we evaluated the prognostic value of the positron emission tomography–computed tomography (PET-CT) imaging technique in patients with newly diagnosed oral squamous cell carcinomas. PET-CT is routinely used to detect and quantify metabolically active tissues such as tumors. By using receiver operating characteristic (ROC) analysis, we successfully determined an optimal cut-off value for patient stratification in order to predict clinical outcome in this population. Furthermore, other clinical variables and their impact on clinical outcome as well as PET-CT values were evaluated. We show that, based on the determined optimal cut-off value, PET-CT is a reliable and independent predictor for clinical outcome, even in a fully adjusted model. Finally, we analyzed mitochondrial DNA to evaluate if potentially deleterious mutations might be a potential cause of metabolic changes, leading to differences in PET-CT values and consequently, clinical outcome.

**Abstract:**

Under aerobic conditions, some cancers switch to glycolysis to cover their energy requirements. Taking advantage of this process, functional imaging techniques such as PET-CT can be used to detect and assess tumorous tissues. The aim of this study was to investigate standardized uptake values and mitochondrial DNA mutations in oral squamous cell carcinoma. A cohort of 57 patients underwent ^18^[F]FDG-PET-CT and standardized uptake values were collected. In 15 patients, data on mitochondrial DNA mutations of the tumor were available. Kaplan–Meier curves were calculated, and correlation analyses as well as univariate Cox proportional hazard models were performed. Using ROC analysis to determine a statistical threshold for SUVmax in PET investigations, a cut-off value was determined at 9.765 MB/mL. Survival analysis for SUVmax in these groups showed a Hazard Ratio of 4 (95% CI 1.7–9) in the high SUVmax group with 5-year survival rates of 23.5% (*p* = 0.00042). For SUVmax and clinicopathological tumor features, significant correlations were found. A tendency towards higher mtDNA heteroplasmy levels in high SUVmax groups could be observed. We were able to confirm the prognostic value of SUVmax in OSCC, showing higher survival rates at lower SUVmax levels. Correlations between SUVmax and distinct tumor characteristics were highly significant, providing evidence that SUVmax may act as a reliable diagnostic parameter. Correlation analysis of mtDNA mutations suggests an influence on metabolic activity in OSCC.

## 1. Introduction

Carcinomas of the oral cavity are among the eleven most common cancers worldwide with an incidence rate of 200,000–350,000 [1]. Although cancers of the oral cavity comprise a wide range of different tumor entities, 95% of all cases are oral squamous cell carcinomas (OSCC) [1]. Several studies were able to identify a multitude of risk factors which—alone or in combination—can cause the development of a malignant disease of the oral cavity [1,2,3]. In particular, tobacco and excessive alcohol consumption as well as infections with human papilloma virus represent major risk factors in industrialized countries, while malnutrition and poor oral hygiene are more often causative in developing countries [1,4]. Due to these circumstances and the significant role of exogenous risk and lifestyle factors, great emphasis is put on prevention, early diagnosis and treatment of this disease. Stage of disease, as determined based on the tumor (T), node (N), and metastasis (M) (“TNM”) classification system, and especially the extent of disease (T) and the presence of metastases (M), show great impact on survival [5]. The overall 5-year survival rate of patients with OSCC in the US is around 60.9% [6]. While prognosis of early stage disease is much better [1], overall survival rates have not improved significantly among this patient collective over recent decades, underlining once again the importance of prevention [7]. Treatment protocols have a great impact on survival rates as well: radically performed surgical interventions show a better prognosis than non-surgical therapies [8]. Treatment options include surgery, radiotherapy, chemotherapy, and combinations of different treatment modalities.

Glycolysis is the key process of cellular glucose breakdown for energy production in eukaryotic cells. Under aerobic conditions, pyruvate is imported into the mitochondria and oxidized to Acetyl-Coenzyme A (Ac-CoA) by the pyruvate dehydrogenase (PDH) complex and finally, is oxidized in the mitochondrial matrix to carbon dioxide during a series of enzymatic steps termed the tricarboxylic acid (TCA) cycle. The resulting electrons are used to drive ATP production by a battery of serially linked enzyme complexes called the electron-transfer system (ETS), during a process called oxidative phosphorylation (OXPHOS). Under anaerobic conditions however, some tissue types and particular tumor types are able to switch to and rely on fermentation, e.g., the production of lactate instead of Ac-CoA from pyruvate, as their main source for ATP generation (aka, the “Warburg” effect) [9]. According to the original Warburg model, complex cellular processes including mutations in genes coding for ETS proteins lead to this metabolic switch [10]. Recent work has extended this model, indicating that not only ETS mutations but also an interplay between various cellular signaling processes and interaction with the tumor microenvironment can lead to this metabolic phenotype [11]. However, metabolic analyses of various tumor models suggest that a fully functional ETS is a prerequisite for the malignant behavior of many cancer types [12].

Proliferating cancer cells are characterized by a high demand of energy to sustain growth and proliferation and energy production by glucose turnover constitutes a main source of ATP [13]. This causes an increased glucose uptake in tumor cells, which can be used for imaging methods such as positron emission tomography (PET) [14]. Currently, the most widely used form of PET is 2-deoxy-2-[^18^F] fluoro-D-glucose (^18^[F]FDG)-PET, where radioactively marked 2-deoxyglucose, is used. Since ^18^[F]FDG cannot be metabolized, it will accumulate in metabolically active tissues as well as tumors and can then be made visible.

The physiological tasks of mitochondria in eukaryotic cells are numerous, encompassing supply of energy, control of redox homeostasis, innate immunity, apoptosis, etc. To accomplish some of these tasks, mitochondria themselves encode some of the necessary genetic information within their own mitochondrial DNA (mtDNA). The mtDNA molecule is a 16.6 kbp small, circular genome located in the mitochondrial matrix encoding for 2 rRNAs, 22 tRNAs, and 13 ETS subunits [15]. While most mtDNA mutations are neutral, thus without any consequence for cellular homeostasis, some variants have been shown to heavily impact cellular energy turnover [16]. Mutations of mtDNA usually emerge as heteroplasmy, which is the presence of two or more mtDNA variants within one mitochondrion or cell, thereby creating a mixture between wild type and variant. In several tumors types, OXPHOS is frequently rewired due to damages of the mitochondrial DNA at functional sites, and some variants are suspected to contribute to malignant transformation [16,17]. In certain instances, these changes might be at the origin of the observed metabolic phenotype by activation of different pathways (such as HIF-activation as a common endpoint) [18], resulting in alternative metabolic traits. Due to these findings, mutations in mtDNA of different tumor entities, including OSCC mutations, have been the object of various studies [19,20,21,22,23].

The primary aim of this study was to evaluate a potential correlation between metabolic tumor activity, as indicated by SUVmax levels with tumor characteristics as well as survival time from the time point of diagnosis. Furthermore, we investigated potential associations between mtDNA mutations in tumor tissue and the metabolic activity as seen by ^18^[F]FDG-PET. Establishing relevant correlations could improve both early detection of the tumor and prognostic predictability.

## 2. Material and Methods

### 2.1. Study Population

In this retrospective, single-center cohort study, patients with histologically verified OSCC between 2005 and 2017 at the Medical University of Innsbruck, Austria, were considered for inclusion. Enrollment of patients was made under local legal and ethical policies. The study protocol was approved by the local ethical committee (EK Nr: 1383/2020).

Pre-existing data were complemented with dates of last follow-up or death. We linked our pre-existing database to an additional validated registry, the Cancer Register Tyrol [24]. This register contains updated data regarding diagnosis, TNM classification, tumor grading and date of death. The routine clinical and radiological follow-up comprised an appointment in our specialized tumor clinic every three months during the first two years with both a clinical examination and a radiological control by sonography or computed tomography. Following this period, intervals were extended to half a year (i.e., two times per year) for an additional three-year period, including a clinical exam and a computed tomography. Finally, patients returned once a year for a clinical exam and a computed tomography from the sixth to tenth year (referring to the completion of initial therapy).

An inclusion criterion was the presence of a primary OSCC in patients who were diagnosed and treated at the Medical University of Innsbruck, Austria. Consequently, patients with recurrent OSCC were excluded. The observation period was determined as the time between diagnosis and last follow-up or death of the patient. Correspondingly, survival rates were defined as the time period between initial diagnosis and date of death or last follow-up. Within the observation period, 30 patients of the study collective died. For the remaining 27 patients, the observation period ended with the last follow-up.

### 2.2. Diagnostic Confirmation

Pathohistological grading from 1 to 4 according to the WHO classification was used to determine the degree of dedifferentiation of tumor tissue [25]. Tumor characteristics including extent of the disease, infiltration of lymph nodes and presence of metastases, were described according to the Union for International Cancer Control (UICC) criteria and staged from I to IV. The presence of bone infiltration was taken from pathological reports and documented qualitatively.

### 2.3. Determination of Standardized Uptake Values

In order to quantify the metabolic activity of tumors in PET-CT imaging, the standardized uptake value (SUV) was used. The SUV is a semiquantitative simplified measurement of the metabolic rate for tissue ^18^[F]FDG uptake. It represents the ratio of the image-derived radioactivity concentration and the whole body concentration of the injected radioactivity. A calibration factor for conversion of the results in MBq/mL is needed which is specific for every camera system.

The SUVs in our study were registered in the hottest areas of the tumors (“SUVmax”) considering individual blood glucose levels and proportion of lean body mass. Therefore, blood glucose levels, body weight and height had to be taken into account prior to measurements. SUVmax was normalized for body weight according to the formula $\mathrm{SUVmax}=\frac{C\left(µ\mathrm{Ci}/\mathrm{mL}\right)}{\frac{\mathrm{ID}\;\left(µ\mathrm{Ci}\right)}{w\left(\mathrm{kg}\right)}}$, where C = activity at a pixel within the tissue, ID = injected dose and w = weight of the patient in kilograms [26]. PET-CT measurements were performed on a Discovery 690 PET-CT machine (GE Healthcare, Milwaukee, WI, USA) and data were visually analyzed using the software program “Hermes SUV SPECT^®^ Reconstructions” (Hermes Medical Solutions, Stockholm, Sweden). To verify test results, PET/CT images were assessed independently by an experienced radiologist (RCM).

To determine the SUVmax cut-off value in regard to survival, ROC curves were generated as well as a Precision–Recall curve. In a first step, the point of highest sensitivity and specificity was identified.

### 2.4. Sequencing of the Entire mtDNA via NGS

To evaluate the mtDNA mutational profile of patients with OSCC, the entire mtDNA was sequenced in paired benign (normal) and malignant (neoplastic) tissue samples by means of NGS as previously reported. This study includes mtDNA data from 15 patients included in a previously published study on OSCC patients for whom SUVmax data were also available [27].

Only mitochondrial DNA samples of patients older than 18 years suffering from histologically proven OSCC were included. For each patient, one benign and one malignant biopsy (2 mm punch needle) were collected directly during tumor resection. These samples were frozen and stored at −80 °C for further use. Following the pathohistological diagnosis, DNA of the tissue samples was extracted on a QIAGEN EZ1 DNA extraction robot using the EZ1 DNA Tissue Kit (Qiagen, Hilden, Germany) according to manufacturers’ instructions. DNA quality and concentration were assessed spectrophotometrically with a TECAN infinite M200 Nanoquant.

The entire mtDNA was amplified by long-range PCR, generating two overlapping fragments as reported previously [21]. After enzymatic fragmentation using the NEBNext dsDNA Fragmentase kit (New England Biolabs, Ipswich, MA, USA)

, dual indexed libraries were produced using the TruSeq Nano DNA HT Kit (Illumina, Inc., San Diego, CA, USA) as previously described [27]. Libraries were sequenced on an Illumina MiSeq instrument, using the MiSeq Reagent Kit v2 (Illumina Inc., San Diego, CA, USA) (500 cycles).

Data analysis was based on FASTQ files, as described previously (see Fendt et al. [27]). Processing of BAM files resulted in the reporting on heteroplasmy frequencies and variants. Quality checks were performed at all levels. The data were analyzed using the mtDNA-Server as previously described [28] by annotating potential artefacts from nuclear encoded mitochondrial pseudogenes (NUMTs: nuclear inserts of mitochondrial genes). Additionally, based on a mixture model comparing different polymerase enzymes [29], we could detect a phantom mutation on position 3210, which was excluded for this analysis.

## 3. Statistics

All statistical analyses were performed using IBM SPSS and R software version 3.5. Using the Kolmogorov–Smirnov test, individual parameters were analyzed regarding normal distribution. Non-normally distributed values were tested using non-parametric, bivariate correlations. The Spearman-Rho test was used to check for statistical significance. ROC curve analysis was performed and visualized in R with the precrec library to determine a statistically valid cut-off value to divide the study population into two groups based on SUVmax data (a high-SUVmax and a low-SUVmax group, respectively). Using non-parametric correlations, the Kaplan–Meier method was used to calculate survival curves while the log-rank test was used to assess statistical differences between survival function. Univariate and multivariate Cox proportional hazard models on overall survival were applied, using SUVmax, bone infiltration, UICC score, grading, gender and age as covariates. Only *p*-values < 0.05 were considered statistically significant.

## 4. Results

### 4.1. Patient Characteristics

Between December 2005 and April 2018, 160 patients with OSCC were treated at the Medical University of Innsbruck, Austria. PET-CT evaluation was performed for 57 of these patients, whereby 23 (40%) of the patients were female, while 34 (60%) were male. UICC stages and grading were available in all patients, and SUVmax values were available in 56 patients who were finally included in the analysis. The average age at initial diagnosis was 63 years (range 32–89 years).

Statistical analysis showed a high prevalence of UICC stage 4 patients equaling 56.1% (32 cases), while the distribution of remaining UICC stages was relatively balanced (stage 1: 10 cases, 17.5%; stage 2: 11 cases, 19.3%; stage 3: 4 cases, 7.0%). No patient had a tumor grading of 4, 8 patients had grading 1, 35 patients had grading 2, and 14 patients had grading 3.

**Figure 1:**
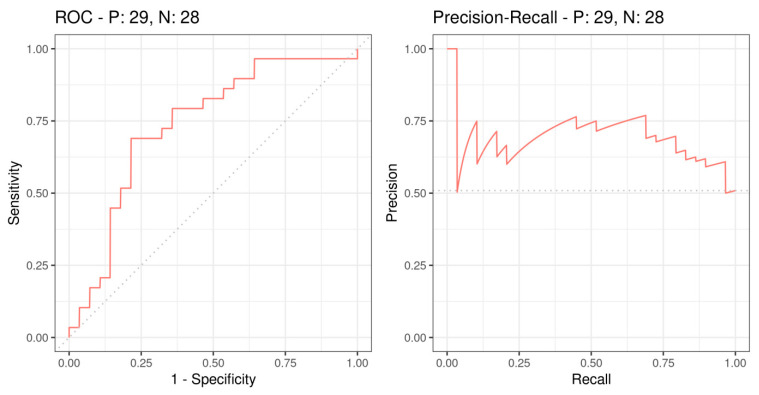
Receiver Operating Characteristic (ROC) and Precision–Recall curves based on survival outcome and SUVmax. P: 29 indicates the 29 deceased patients, N: 28 indicates the 28 living patients.

In our study, the cut-off value for SUVmax based on ROC analysis was 9.765 MB/mL which corresponds to a sensitivity of 69.0% and a specificity of 78.8%. The AUC was 0.76 and the corresponding *p*-value was 0.002. ROC analysis and Precision-Recall curves are shown in Figure 1.

The average SUVmax value of the study group was 10.63 MBq/mL (range, 1.65–26.62 MBq/mL). There was no statistical difference in SUVmax between female and male patients (Figure 1). In one patient, data on SUVmax and bone infiltration were missing. Distribution of grading within the different SUVmax groups was relatively balanced, and no statistically significant difference was found (*p* = 0.262, Chi-squared test). Distribution of UICC classification within the SUVmax groups showed that patients with lower tumor stages (staging 1 and 2) were associated with lower SUVmax values, whereas patients with higher stages (3 and 4) were significantly associated with higher SUVmax values (Chi-squared test, *p* = 0.012).

### 4.2. Correlation of Clinical Parameters

Table 1 shows the level of significance, the correlation coefficient, and the number of cases used for correlations. There is a statistically significant correlation between SUVmax, grading (*p*-value = 0.028), UICC stage (*p*-value = 0.003) and bone infiltration (*p*-value < 0.001). The correlation between UICC and grading was found to be highly significant (*p* < 0.001). All other parameters have not shown significant correlations.

**Figure 2:**
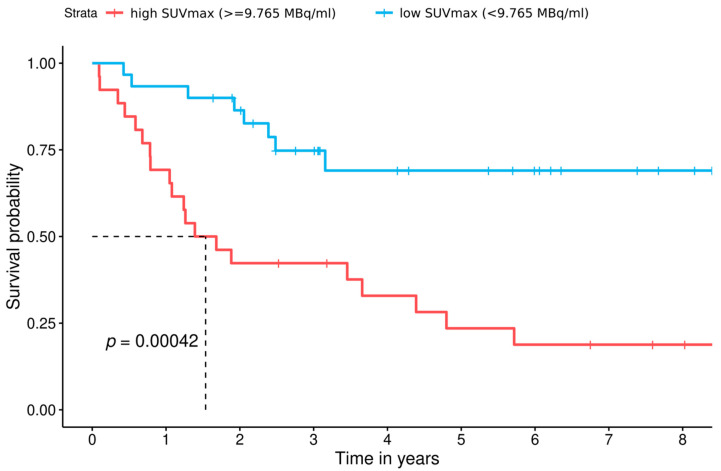
Survival functions of both SUVmax groups discerned by the cut-off value (low SUVmax group: <9.765 MB/mL, blue line; high SUVmax group: ≥9.765 MB/mL, red line). In the low SUVmax group, 8 patients died (5-year survival of 69%), while in the high SUVmax group, 20 patients died (corresponding to a 5-year survival rate of 23.5%). The overall survival rates were 69% in the low and 18.8% in the high SUVmax groups, respectively.

In 56 of 57 patients, SUVmax values were collected from pre-existing PET-CTs. In 1 out of the 57 patients, PET-CT was not accessible for SUVmax analysis. The remaining 56 patients were divided into two groups using the cut-off value (<9765 MB/mL = 0, *n* = 30; ≥9.765 MB/mL = 1, *n* = 26) to calculate survival functions (Figure 2).

**Figure 3:**
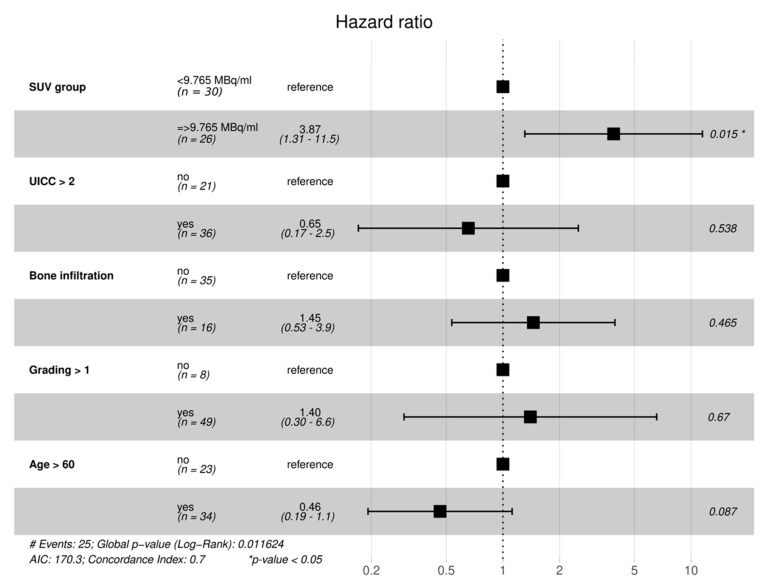
Fully adjusted model–SUVmax group still shows significantly higher hazard-ratio, after adjusting for UICC, bone infiltration status, Grading and age above 60.

**Figure 4:**
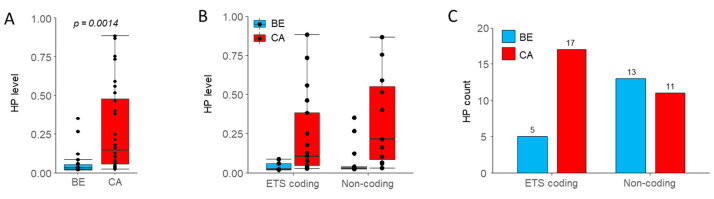
(**A**) Heteroplasmic levels in all 15 benign and malignant tissue samples. Significantly higher levels of heteroplasmies in OSCC (*p* = 0.0014). (**B**) Heteroplasmic levels and (**C**) heteroplasmy counts in either ETS or non-coding regions of the mtDNA in both SUVmax groups. No statistically significant difference between low (*n* = 7) and high SUVmax groups (*n* = 8) could be observed with the Wilcoxon test.

Analysis of overall survival in the two subgroups showed a statistically significant difference between the two groups (*p* = 0.00042). Median overall survival was 561 days (~1.5 years) in the high SUVmax group and 1118.5 days (~3 years) in the low SUVmax group (HR = 4, *p* = 0.0011). The 5-year survival rate of the low SUVmax group was 69% in comparison to 23.5% of the high SUVmax group. Overall survival was similar in women and men (Figure 2) and, when stratified for PET-CT activity, overall survival was shorter in patients exhibiting high SUVmax levels irrespective of gender, indicating the same trend. However, the impact of high SUVmax levels on overall survival was more pronounced in female patients (Figure 3) when compared to male patients (Figure 4).

Table 2 shows the results of the univariate Cox regression analysis of distinct patient and tumor characteristics on survival rates. Only high SUVmax levels and age above 60 years have a significant impact on survival. Consequently, high SUVmax levels are associated with lower survival rates, and age above 60 years with higher survival rates.

In a fully adjusted model (Figure 3), SUVmax group still proved to have a statistically significant impact on survival in our study collective (*p* = 0.015).

### 4.3. MtDNA Mutational Profile in High vs. Low SUVmax Groups

In order to evaluate if the observed metabolic traits might be associated with the presence of mtDNA mutations, we sequenced the entire mtDNA of paired benign and malignant tissue in a subset of 15 patients by NGS (low-SUVmax: *n* = 7, high-SUVmax: *n* = 8). An in-depth analysis on mtDNA mutations in the entire prospective cohort has recently been published [27]. Applying a threshold of 2% for the heteroplasmy detection, which after performing in-depth analysis has been shown to produce results less prone to artefacts [29], 51 mutations were detected overall (46 private and 5 shared). Heteroplasmies (HP) were found in 11 of the benign samples and 10 of the malignant samples and, in concordance with the overall cohort, private mutations in OSCC samples were more frequent compared to the paired benign tissue (*n* = 28 vs. 18). Additionally, we detected significantly higher heteroplasmic levels in OSCC compared to benign tissue samples (Figure 4A, *p* = 0.00014, Wilcoxon rank-sum test). In our population, the median heteroplasmy level was 17.1% in OSCC and 3.3% in benign tissue, respectively, with similar differences between ETS-coding and non-coding regions (Figure 4B). Additionally, mutation rates in OSCC samples were significantly higher in ETS coding regions compared to the corresponding benign tissue (Figure 4C, *p* = 0.038, Fisher’s exact test). These findings are in line with the complete dataset (see Fendt et al. [27])

**Figure 5:**
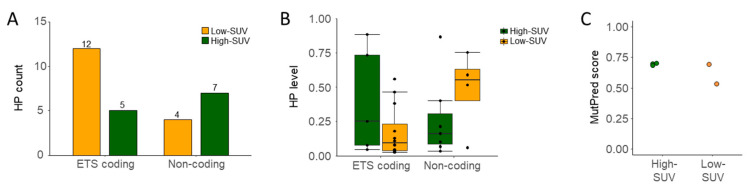
(**A**). Heteroplasmic levels in all malignant tissue samples (*n* = 15) only. (**A**) Heteroplasmy count and (**B**) heteroplasmy levels in low vs. high SUVmax subgroups. No statistically significant difference between low (*n* = 8) and high SUVmax groups (*n* = 7) could be observed. (**C**) MutPred pathogenicity score of mutations leading to amino-acid alterations in high- vs. low-SUVmax subgroups.

When looking at mutation profiles according to SUVmax groups, no statistically significant differences in terms of HP numbers and/or levels have been detected. Overall, 12 mutations were found in the high-SUVmax group and 16 in the low-SUVmax group, respectively, while median HP levels were 18.8% and 11.7% in the high- vs. low-SUVmax subgroup. HP numbers and levels were similar in both groups in non-coding regions but, more importantly, also in the ETS coding regions of the mtDNA (Figure 5A,B). In the high-SUVmax subgroup, 5 out of 8 samples (62.5%) harbored mutations in ETS genes compared to 4 out of 7 samples (57.1%) in the low-SUVmax group. While the number of mutations located in the ETS genes tended to be numerically higher in the low-SUVmax subgroup, the results did not reach statistical significance (Figure 5A). Additionally, HP levels in ETS or non-coding regions were not statistically different between the SUVmax subgroups (Figure 5B). When looking at samples with mutations in ETS genes leading to an amino acid change with likely effect on protein function and a high enough mutation level (>20%), three patients in the high-SUVmax group (37.5%) and two patients in the low-SUVmax group (28.6%) were affected (Figure 5C). Additionally, the likely effect on protein function as indicated by MutPred score [30] appears similar. These findings indicate that differences in metabolic profiles are likely not the result of mutations in mtDNA ETS genes or mtDNA mutations in general.

## 5. Discussion

The prognostic value of tumor characteristics such as TNM classification or grading has been clinically well-established and proven through numerous studies [7,31,32,33,34]. However, other prognostic parameters are still of limited clinical relevance and use. Modern imaging techniques such as PET-CT use metabolic adaption processes in tumors to detect the presence of neoplastic tissues and might thus be of use for advanced patient stratification regarding prognostic and therapeutic evaluation. Studies on different tumor entities confirmed the feasibility to assign a predictive value to this metabolic activity quantified by the SUVmax, measured in ^18^[F]FDG PET and PET-CT. The method has shown to represent a valid marker for prognosis, recurrence and survival time [35,36,37,38,39,40,41,42,43,44,45,46,47]. Berghmans et al. also highlighted this connection early in their review of the predictive value of SUVmax on survival times in patients with non-small-cell lung cancer (NSCLC) and identified a survival-dependent threshold for SUVmax [44]. In further studies on OSCC, the same correlations were observed [36,48,49,50,51]. Based on these pre-existing studies, we evaluated a potential survival-dependent impact of SUVmax for OSCC patients and established a tumor-specific threshold by use of ROC analyses. Furthermore, we evaluated potential correlations between the metabolic activity and certain tumor characteristics.

In line with previous studies demonstrating a correlation between metabolic activity, survival and clinicopathological features in OSCC [48,49,51,52], we were able to confirm these findings in the present study. Analysis of SUVmax values among patients with OSCC—divided into two groups according to a statistically calculated cut-off value—showed highly significant differences in survival, with higher survival rates in the low SUVmax group. According to our results, our initial assumption that the metabolic activity in OSCC might have an impact on survival was confirmed, and evidence for an SUVmax-dependent behavior of tumor characteristics was demonstrated. In our study collective, significant correlations between metabolic activity and clinicopathological tumor features were seen. Correlation analysis demonstrated significant association between SUVmax and grading as well as UICC. These results indicate a dependency between the local metabolic activity of tumors as seen by PET-CT and histopathological characteristics of the tumor, which are consistent with previous results [35]. According to Stalder et al. [52], we also found a strong relationship between SUVmax and the presence of bone infiltration in our study collective [52]. These results, as well as the statistically significant correlation between grading and UICC, indicate that undifferentiated tumors also tend to grow faster and more aggressively with respect to their metabolic activity [38,39,47]. Prior results also showed higher mortality rates among patients with higher grading and higher tumor stage (4) as well as a higher occurrence of bone infiltration in tumors with higher metabolic activity in PET-CTs [52].

Similar to previous studies, we defined a statistically reliable SUVmax threshold for discriminating our study collective into two groups—a high- and a low SUVmax group. By using ROC-analysis, we found the cut-off value for SUVmax at 9.765 MBq/mL in our population. In a period of five years, the low SUVmax group showed a statistically significantly longer survival than the high SUVmax group. The data allowed for a clear distinction of both groups, confirming the results of the mentioned studies. Even after adjusting models for possible confounding factors, survival analysis showed significant results, confirming the prognostic value of SUVmax. In prior studies, cut-off values were defined as well, resulting in a broad range of SUVmax thresholds between 4.76 and 19.3 MBq/mL due to a lack of standardization [49]. Furthermore, age above a certain threshold (in our study population, 60 years) seems to have a positive impact on survival. Preliminary studies confirm this relationship for certain tumor entities such as colon and breast cancer, assuming higher tumor aggressiveness in young people due to a lack of screening, certain driver mutations or hormonal influences [53]. Due to the limited number of cases however, we have to emphasize that further studies in larger cohorts are needed to test and verify our findings.

The impact of mitochondrial DNA genetics on tumor cell proliferation and malignant transformation is still being investigated [54], but several studies have shown a link between mtDNA mutations and the clinical phenotype in certain cancer types [55] as well as OSCC [27]. Defects in ETS genes coding for functional sites have shown to lead to subsequent changes in the metabolic phenotype of cancer cells [16]. However, further factors may also contribute to these metabolic changes and, since mutations in the mtDNA usually present as heteroplasmic variants, a certain mutation threshold must be surpassed, before changes in cellular energy metabolism take place [27,56]. Sixty-four years ago, Otto Warburg proposed his hypothesis of metabolic transformation in cancer cells, which is based on potential dysfunctions in ETS proteins, leading to a switch from OXPHOS to fermentation of glucose, even under normoxic conditions [10]. Since then, research has shown that tumor metabolism is not a static function but rather an essential part of metabolic reprogramming based on necessary adaptations caused by external or internal cellular factors [57]. It has been shown for example that the interplay between hypoxia-inducible factor-1 (HIF-1) overexpression, HIF-1 cooperation with epigenetic mechanisms, oncogene activation (cMyc, Ras), loss of function of tumor suppressors (mutant p53, mutant PTEN, miRNAs and sirtuins with suppressor functions), activated (PI3K-Akt-mTORC1, Ras-Raf-MEK-ERK-cMyc, Jak-Stat3) or deactivated (LKB1-AMPK) signaling pathways, and components of the tumor microenvironment (such as cancer-associated-fibroblasts) are likely to influence the metabolic phenotype of a tumor cell [11]. To see if the metabolic reprogramming of OSCC cells leading to increased SUVmax value might be linked to dysfunctions in ETS proteins, we evaluated if mutations in mtDNA coding for ETS genes at a certain level of heteroplasmy could have effects on SUVmax values by alterations in energy metabolism. In line with recent studies [12], our study was also not able to confirm this hypothesis since the rate for potentially deleterious mtDNA mutations leading to significant amino acid substitutions in ETS proteins in both the high- and low-SUVmax groups were similar. This again highlights that usually, a fully functional ETS is a prerequisite for tumor growth and malignant behavior, while other factors such as oxygen deprivation, oncogene activation and the tumor microenvironment are likely to cause the observed shift in the malignant metabolic behavior [58]. However, since we only analyzed mtDNA mutations in a small proportion of patients undergoing PET-CT analysis, additional research is needed to fully unravel the potential association of mtDNA alterations and SUVmax. Recent research also challenges the concept of the Warburg effect being the driving force for increased glucose uptake as determined by ^18^[F]FDG-PET in many cancer types, favoring the notion that limited oxygen availability and consequently, the Pasteur effect, might be the major contributor leading to this metabolic switch [59].

This work has limitations to be considered when interpreting the results. With a total of 57 patients and 15 patients with data on mtDNA mutations, the study size is relatively small. Consequently, statistical calculations (especially in subgroups) often fail to achieve a sufficiently high statistical power. Due to the retrospective nature of this work, missing data are an inherent shortcoming of this study type. The time interval between PET-CT and date of initial diagnosis could not be set uniformly. Variable duration of time intervals occurred. Additionally, the very limited number of sequenced mtDNA genomes in this study limits the power of the analyses. Furthermore, while ^18^[F]FDG-PET certainly detects metabolic changes and alterations in glucose uptake of tumor cells with respect to the tissue of origin and/or surrounding tissue, the main limitation of this technology is that it is not able determine the eventual catabolic fate of glucose once it enters the cell. Functional studies involving enzyme activities and protein expression are needed to investigate the precise mechanism, leading to extended glucose uptake in OSCC tissue with high-SUVmax to yield further diagnostic and potentially therapeutic advantages.

## 6. Conclusions

In summary, we were able to confirm the importance of 18FDG-PET evaluation as a valuable diagnostic and prognostic tool in patients with OSCC. Furthermore, the correlations between survival and clinicopathological tumor features in this study support our initial hypothesis and provide an understanding of the SUVmax-dependent survival rates above a determined threshold and its dependency on tumor characteristics. Analyses of mtDNA in a subset of our patients were able to gain initial results on a potential linkage of mutations and clinical course in OSCC.

## Figures and Tables

**Table 1:** Spearman-Rho correlation between different parameters.

Variable	Variable Characteristics	UICC	Grading	SUVmax	Bone Infiltration
UICC	Correlation coefficient	1.000	0.572	0.390	0.531
	Sig. (bilateral)		0.000	0.003	0.000
	Cases	57	57	56	51
Grading	Correlation coefficient	0.572	1.000	0.294	0.264
	Sig. (bilateral)	0.000		0.028	0.61
	Cases	57	57	56	51
SUVmax	Correlation coefficient	0.390	0.294	1.000	0.382
	Sig. (bilateral)	0.003	0.028		0.006
	Cases	56	56	56	51
Bone Infiltration	Correlation coefficient	0.531	0.264	0.382	1.000
	Sig. (bilateral)	0.000	0.061	0.006	
	Cases	51	51	51	51

Overall survival in pre-defined SUVmax groups.

**Table 2:** Univariate Cox proportional-hazard models for different patient and tumor characteristics. *p*-values < 0.05 are considered statistically significant. HR = Hazard Ratio with 95% Confidence Interval.

VARIABLE	HR (95% CI)	*p*-VALUE
HIGH SUV_MAX_	4.0 (1.7–9)	0.0011
AGE ≥ 60	0.44 (0.21–0.94)	0.033
BONE_INFILTRATION	2.2 (1–5)	0.051
UICC ≥ 3	2.1 (0.91–5)	0.08
GRADING ≥ 2	1.6 (0.49–5.4)	0.43
FEMALE GENDER	0.98 (0.46–2.1)	0.95

## Data Availability

The data presented in this study are available upon reasonable request from the corresponding author.
